# Identifying sex-specific anthropometric measures and thresholds for dysglycemia screening in an HIV-endemic rural South African population

**DOI:** 10.1371/journal.pgph.0001698

**Published:** 2023-10-27

**Authors:** Alison C. Castle, Susanne S. Hoeppner, Jennifer M. Manne-Goehler, Stephen Olivier, Itai M. Magodoro, Urisha Singh, Johnathan A. Edwards, Frank Tanser, Ingrid V. Bassett, Emily B. Wong, Mark J. Siedner, on behalf of the Vukuzazi Study Team

**Affiliations:** 1 Africa Health Research Institute, KwaZulu-Natal, South Africa; 2 Division of Infectious Diseases, Massachusetts General Hospital, Boston, Massachusetts, United States of America; 3 Harvard Medical School, Boston, Massachusetts, United States of America; 4 Emory Global Diabetes Research Center, Rollins School of Public Health, Emory University, Atlanta, Georgia, United States of America; 5 University of KwaZulu-Natal, Durban, KwaZulu-Natal, South Africa; 6 Department of Biostatistics and Bioinformatics, Emory University, Atlanta, Georgia, United States of America; 7 School of Health and Social Care, University of Lincoln, Lincoln, United Kingdom; 8 Lincoln Institute for Health, University of Lincoln, Lincoln, United Kingdom; 9 Division of Infectious Diseases, University of Alabama Birmingham, Birmingham, Alabama, United States of America; PLOS: Public Library of Science, UNITED STATES

## Abstract

Valid screening and diagnostic algorithms are needed to achieve 2030 targets proposed by the WHO’s *Global Diabetes Compact*. We explored anthropometric thresholds to optimally screen and refer individuals for diabetes testing in rural South Africa. We evaluated screening thresholds for waist circumference (WC), body mass index (BMI), and waist-hip ratio (WHR) to detect dysglycemia based on a glycated hemoglobin (HbA1C) ≥6.5% among adults in a population-based study in South Africa using weighted, non-parametric ROC regression analyses. We then assessed the diagnostic validity of traditional obesity thresholds, explored optimal thresholds for this population, and fit models stratified by sex, age, and HIV status. The prevalence of dysglycemia in the total study population (n = 17,846) was 7.7%. WC had greater discriminatory capacity than WHR to detect dysglycemia in men (p-value<0.001) and women (p<0.001). WC had greater discriminatory capacity than BMI to detect dysglycemia in women (p<0.001). However, BMI and WC performed similarly for men (p = 0.589). Whereas traditional WC thresholds for women (>81cm) performed well (sensitivity 91%, positive predictive value [PPV] 14.9%), substantially lower thresholds were needed to achieve acceptable sensitivity and PPV among men (traditional >94cm, derived >79.5cm). WC outperforms BMI as an anthropometric screening measure for dysglycemia in rural South Africa. Whereas WC guideline thresholds are appropriate for women, male-derived WC cutoffs performed better at lower thresholds. In this rural South African population, thresholds that maximize specificity and PPV for efficient resource allocation may be preferred.

## Introduction

Approximately 80% of the estimated 537 million adults with diabetes worldwide live in low- and middle-income countries [1]. In Sub-Saharan Africa, more than half of those with type 2 diabetes are undiagnosed and unaware of their condition [1]. To purposefully respond to this growing epidemic, the World Health Organization (WHO) launched the *Global Diabetes Compact* in April 2021 with a focus on improving care for diabetes in low- and middle-income countries [2]. One of the global targets proposed by this initiative includes diagnosing 80% of adults living with diabetes by 2030, a target which based on current data remains far out of reach [3–5]. The feasibility of population-wide diabetes testing remains unclear [6]. Moreover, in settings with overburdened healthcare systems, such as South Africa, cost effective strategies to screen and diagnose individuals with diabetes are needed.

Anthropometric measures including body mass index (BMI) and waist circumference (WC) are recommended as screening modalities to identify those with metabolic syndrome [7,8]. In practice, weight and body mass index are the most commonly used measures in South Africa and are recommended as part of the primary healthcare guidelines for diabetes screening [9]. By contrast, WC is rarely collected outside of research studies, despite its relative ease of capture and feasibility in resource-limited settings. Alternative measures that reflect abdominal adiposity, including WC and waist-hip ratio (WHR), have been shown in some populations to be better indicators of diabetes risk than BMI [10,11].

Optimal anthropometric thresholds to trigger additional screening also remain a matter of debate. The WHO and the International Diabetes Federation (IDF) recommend region-specific thresholds because the relationship between obesity and metabolic disease varies depending on ethnicity and geographic context [7,12]. In sub-Saharan Africa, local clinical guidelines still recommend thresholds derived from European and Western populations; however, this is not well supported empirically [13–17]. For example, three recent studies in South Africa found that derived WC thresholds for metabolic syndrome differ from those recommended by the International Diabetes Federation [17–19]. These differences may be in part due to the high prevalence of human immunodeficiency virus (HIV) within South Africa, where as many as 1 in 5 adults between the ages of 15–49 years are living with HIV [20]. HIV is independently associated with dysglycemia and those on antiretroviral therapy are at risk for excessive weight gain, further complicating the relationship between obesity and diabetes [21]. As such, anthropometric thresholds for BMI, WC, and WHR that optimally stratify populations in sub-Saharan Africa for targeted screening of diabetes are urgently needed.

We assessed the performance of anthropometric measures and their thresholds to detect dysglycemia in a large, population-based study in rural KwaZulu-Natal, South Africa. Due to the cross-sectional nature of this study and because diabetes diagnoses require repeated testing over time, we used the term dysglycemia to indicate a hemoglobin A1c greater than or equal to 6.5% [22]. We compared the discriminatory performance of BMI, WC, and WHR for the detection of dysglycemia. We then assessed the validity of traditionally defined thresholds to detect prevalent disease and, where appropriate, derived new thresholds to augment the diagnostic validity of dysglycemia screening in South Africa.

## Methods

### Study design and setting

We analyzed data from the Vukuzazi Study, a population-sampled, cross-sectional study that enrolled residents in the uMkhanyakude District of KwaZulu-Natal, South Africa. Full study procedures have been reported previously [23]. Briefly, all adolescent and adult resident members of the Africa Health Research Institute Demographic Health Surveillance Site greater than 15 years were visited at home and invited to participate in a mobile health fair that traveled through the study catchment area between May 2018—November 2019. Of the 34,721 eligible participants in the region, 24% (n = 8261) were unable to be contacted, and 2% refused to attend the health screening (n = 862). Ultimately 18,041 attended and enrolled into the parent study, which includes 51.9% of those eligible. The population in this region of South Africa has approximately 100% of individuals of Black African descent, 58% of adults unemployed, and 66% with access to piped water in their home [24]. Participants completed questionnaires on sociodemographic measures, medical history, and medications pertaining to diabetes and HIV serostatus. Venipuncture whole blood samples were collected for hemoglobin A1c (VARIANT II TURBO Hemoglobin test system (Bio-Rad, Marnes-la-Coquette, France) and HIV (Genscreen Ultra HIV Ag-Ab enzyme immunoassay (Bio-Rad)). Participants with a positive HIV immunoassay had a reflex HIV-1 RNA viral load performed (Abbott RealTime HIV-1 Viral Load, Abbott, Illinois, USA).

### Study definitions

Our predictors of interest were the anthropometric measures BMI, WC, and WHR. Anthropometrics were measured by study enrolled nurses and obtained according to the WHO STEPwise Approach to Non-communicable disease Risk Factor Surveillance protocol [25]. Participants were asked to remove footwear, heavily weighted hairdos, belts, and pocket items before standing on the scale with heels against the labeled board for height and weight. WC was measured directly on the skin, or when not possible over light clothing, at the midpoint between the lower margin of the last palpable rib and the top of the iliac crest, at the end of normal expiration. Hip circumference was taken around the widest portion of the buttocks. We defined these measures using the following factors: 1) weight, measured in kilograms (kg); 2) WC, measured in centimeters; 3) hip circumference, measured in centimeters 4) WHR; and 5) BMI, which was calculated as weight in kg divided by the square of height in meters (kg/m^2^).

Our primary outcome of interest was dysglycemia. The presence of dysglycemia was defined on the basis of current WHO diagnostic thresholds as a hemoglobin A1c ≥6.5% (48.0 mmol/mol) [26]. Participants who reported use of medication for Type 2 diabetes within the last two weeks were also classified as having dysglycemia, irrespective of biomarker values. Participants with a positive HIV immunoassay were defined as having HIV. Controlled HIV disease was characterized as having an HIV-1 RNA viral load of ≤ 40 copies per mL in participants who reported antiretroviral use, whereas those with a viral load > 40 copies per mL were considered to have uncontrolled HIV.

### Statistical methods

We used inverse probability of sampling weights to account for study non-attendance and estimate population-level characteristics for all residents within the region. To do so, we first fitted logistic regression models among all resident adults aged >15 years in the Africa Health Research Institute Demographic and Health Surveillance Site with attendance at the Vukuzazi Health Fair as the outcome of interest [27]. Predictors included 5-year age bands and sex. The inverse of the predicted probabilities to attend the Vukuzazi Health Fair from the regression model were used as weights in subsequent analyses, including estimating summary statistics to describe the sample. Enrolled Vukuzazi participants who completed all anthropometric measurements and hemoglobin A1c testing were included in this analysis.

We conducted all sample descriptive and primary analyses stratified by sex because guidelines for most anthropometric measurements, including WC and WHR, are sex-specific [25]. To compare the diagnostic validity of each anthropometric measure, we constructed receiver operating characteristic (ROC) curves, estimated the area under the curve (AUC), and performed weighted parametric regression analyses of ROC curves using maximum likelihood. We then calculated the sensitivity, specificity, and positive predictive value of applying traditional thresholds of overweight and obesity using the following definitions: BMI was classified into categories as recommended by the WHO: underweight (<18.5 kg/m^2^), normal (18.5–24.9 kg/m^2^), overweight (25.0–29.9 kg/m^2^), and obesity (≥30.0 kg/m^2^) [28]. Similarly, WC (National Heart Lung Blood Institute (NHLBI) [29]: >102 cm men, >88 cm women; IDF: >94 cm men, >80 cm women) and WHR thresholds (>0.9 cm men, >0.85 cm women) were classified according to the WHO Waist Circumference and Waist-Hip Ratio Report [30]. After determining that traditional thresholds had substantially lower sensitivities for men, we proceeded to explore alternative thresholds for WC that would provide a sensitivity >90%, specificity >60%, or an optimized Youden index calculation (Sensitivity + Specificity– 1) to detect dysglycemia [31].

Finally, we sought to explore whether relationships between anthropometric measures and the prevalence of dysglycemia differed by key demographic factors and HIV status. To do so, we first fit univariate logistic regression models for dysglycemia between each anthropometric measure, and then adjusted the models for sex, age strata (<30 years, 30–50 years, >50 years), and HIV categories (HIV negative, HIV controlled, HIV uncontrolled) (S1 Table). We included the pre-specified covariates of age and sex in our multivariable models as these are known confounders for anthropometric-dysglycemia relationship [32,33]. We then included two-way interactions terms between each anthropometric measure and sex, age strata, and HIV categories, respectively (S2 Table). Statistical analyses were conducted in Stata (Version 17, Statacorp, College Station, Texas USA).

### Ethical considerations

The Vukuzazi study was approved by the institutional review boards at the University of KwaZulu-Natal Biomedical Research Ethics Committee (BE560/17) and Mass General Brigham (2018P001802). All participants gave written informed consent to participate.

## Results

Of the eligible adults within the catchment area (n = 34,721), 18,041 (51.9%) were enrolled in the Vukuzazi study. A total of 17,846 (98.9%) completed all anthropometric measurements, had HbA1C testing results and were included in this analysis. Women (OR 2.03 [95% CI 1.94–2.12]; p<0.001) and individuals over the age of 50 (OR 2.01 [95% CI 1.92–2.11]; p<0.001) were more likely to attend the Vukuzazi health screenings compared to men and younger participants in the region.

The estimated population prevalence of dysglycemia was 7.7% (Table 1). Those living with HIV accounted for 33.8% of the population, with women having a higher prevalence of HIV compared to men (40.2 vs. 24.6%) Based on WHO thresholds, 50.9% of individuals had a body mass index above normal (BMI ≥25 kg/m^2^), and 29.2% would be considered to have obesity (BMI ≥30 kg/m^2^). Applying the NHLBI thresholds for WC, 6.3% of men and 54.3% of women would screen positive for dysglycemia using a WC > 102 cm in men and a WC > 88 cm in women. Using the International Diabetes Federation thresholds, a greater percentage of the population met criteria for confirmatory diabetes testing (Table 1).

**Table 1:** Estimated weighted baseline population characteristics.

Characteristics	Total Population	Normoglycemia		Dysglycemia	
		Men	Women	Men	Women
Number	17,846	5,451	10,795	292	1,330
Proportion (%)	100%	30.5	60.5	1.6	7.5
Sex (%)	N/A	42.7	57.3	23.3	76.7
Age (years)	37.5 (37.3–37.7)	32.7 (32.3–33.1)^†††^	38.3 (38.0–38.7)^†††^	54.0 (51.9–56.1)^†††^	58.0 (57.2–58.8)^†††^
Living with HIV (%)	33.8	24.7	42.0^†††^	23.2	76.8^†††^
Controlled HIV (%)	80.6	70.7^††^	84.3^†^	89.4^††^	88.2^†^
Mean Weight (kg)	71.0 (70.8–71.3)	65.8 (65.4–66.2)^†††^	73.0 (72.6–73.3)	81.1 (78.7–83.4)^†††^	86.6 (85.5–87.6)
Mean Height (cm)	163.0 (162.9–163.2)	168.8 (168.6–169.0)	159.1 (159.0–159.2)^†††^	168.0 (167.0–169.0)	158.0 (157.6–158.4)^†††^
**BMI (kg/m2)**					
Mean BMI	26.9 (26.7–27.0)	23.1 (23.0–23.2)^†††^	28.8 (28.7–28.9)^†††^	28.5 (27.8–29.3)^†††^	34.7 (34.2–35.1)^†††^
*Estimated % of Population*					
WHO* ≥ 25	50.9 (50.1–51.6)	25.8 (24.6–27.0)	64.6 (63.7–65.5)	71.8 (66.3–76.9)	91.7 (90.1–93.1)
WHO* ≥ 30	29.2 (28.5–29.8)	8.5 (7.7–9.2)	39.6 (38.7–40.5)	39.6 (34.0–45.5)	73.2 (70.7–75.5)
**Waist Circumference (cm)**					
Mean Waist Circumference	85.8 (85.6–86.1)	78.7 (78.4–79.0)^†††^	89.0 (88.7–89.3)^†††^	93.3 (91.4–95.3)^†††^	103.9 (103.1–104.8)^†††^
*IDF Thresholds* ^*^*^					
Men > 94 cm	N/A	10.3 (9.5–11.1)	N/A	50.9 (45.1–56.8)	N/A
Women > 80 cm	N/A	N/A	67.5 (66.6–68.4)	N/A	91.7 (90.1–93.1)
*NHLBI Thresholds* ^‡^					
Men >102 cm	N/A	9.5 (9.5–9.6)	N/A	31.5 (26.4–37.1)	N/A
Women > 88 cm	N/A	N/A	49.0 (48.1–50.0)	N/A	84.9 (82.9–86.8)
**Waist Hip Ratio**					
Mean Waist Hip Ratio	0.848 (0.847–0.849)	0.853 (0.851–0.855)^†††^	0.835 (0.834–0.837)^†††^	0.923 (0.911–0.935)^†††^	0.910 (0.904–0.915)^†††^
*Estimated % of Population*					
WHO^§^ Men > 0.9	N/A	23.6 (22.5–24.7)	N/A	57.3 (51.3–63.0)	N/A
WHO^§^ Women > 0.85	N/A	N/A	42.3 (41.4–43.3)	N/A	75.0 (72.6–77.3)

Weighted values reported as mean (95% confidence interval) or percentage.

^†^ p<0.05

^††^ p<0.01

^†††^p<0.001, for comparisons between Normoglycemia versus Dysglycemia within a specific gender.

*World Health Organization (WHO) thresholds for overweight and obese (≥25 kg/m^2^) and obese only (≥30 kg/m^2^).

^^^ International Diabetes Federation (IDF) waist circumference thresholds for cardiometabolic disease risk in men (>94 cm) and women (>80 cm).

^‡^ National Heart, Lung, Blood Institute (NHLBI) waist circumference thresholds for cardiometabolic disease risk in men (>102 cm) and women (>88 cm).

^§^ WHO waist-hip ratio thresholds for cardiometabolic disease risk in men (>0.9) and women (>0.85) cm (centimeters); kg (kilograms); HIV (human immunodeficiency virus).

**Fig 1:**
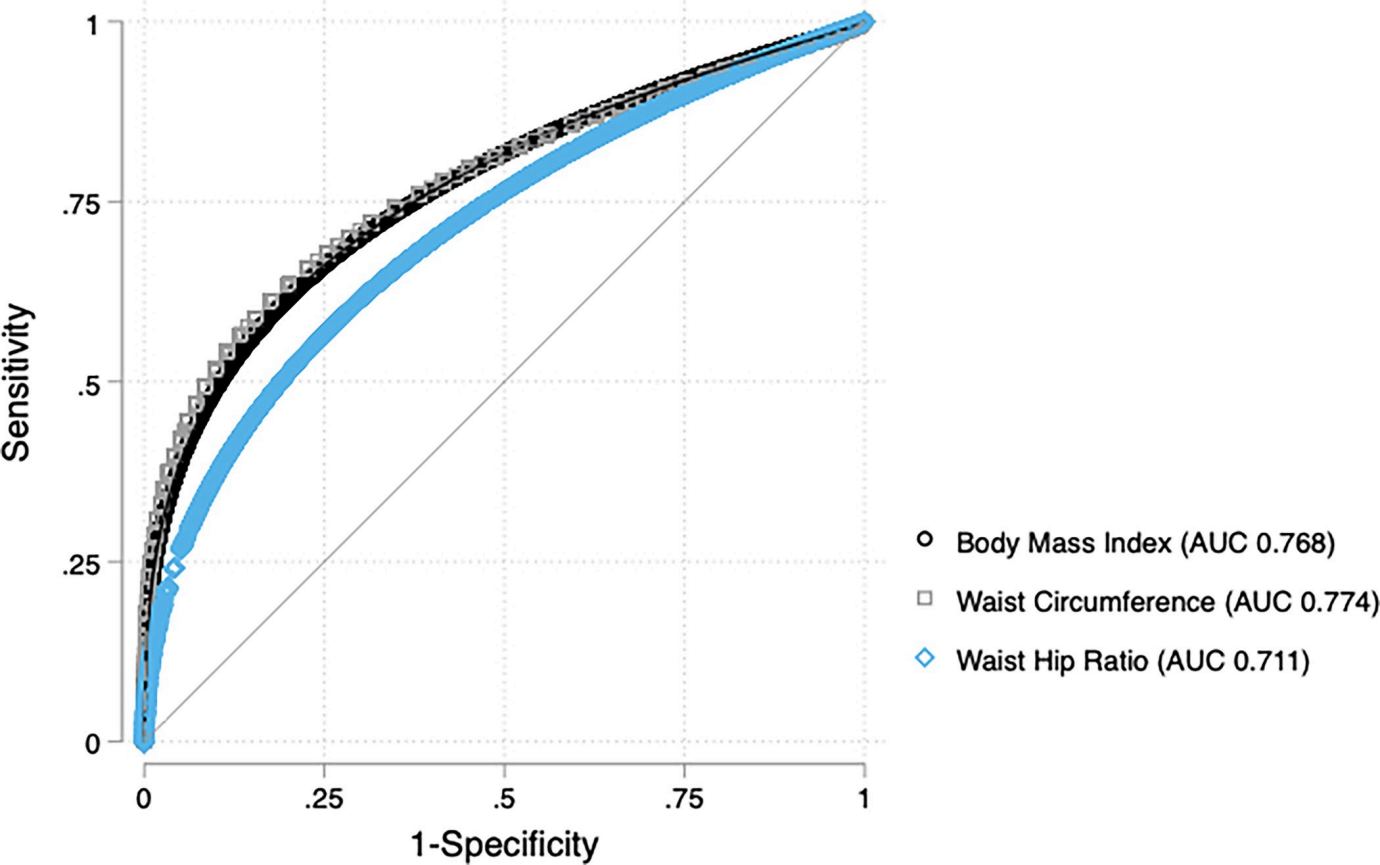
Anthropometric ROC curves for dysglycemia in men. Receiver Operating Characteristic (ROC) Curves for Body Mass Index, Waist Circumference, and Waist Hip Ratio to detect dysglycemia. In men, the area under the curve (AUC) for each anthropometric measure was 0.768 for BMI (95% CI 0.739–0.797), 0.774 for Waist Circumference (95% CI 0.744–0.805), and 0.711 for Waist Hip Ratio (95% CI 0.678–0.744).

**Fig 2:**
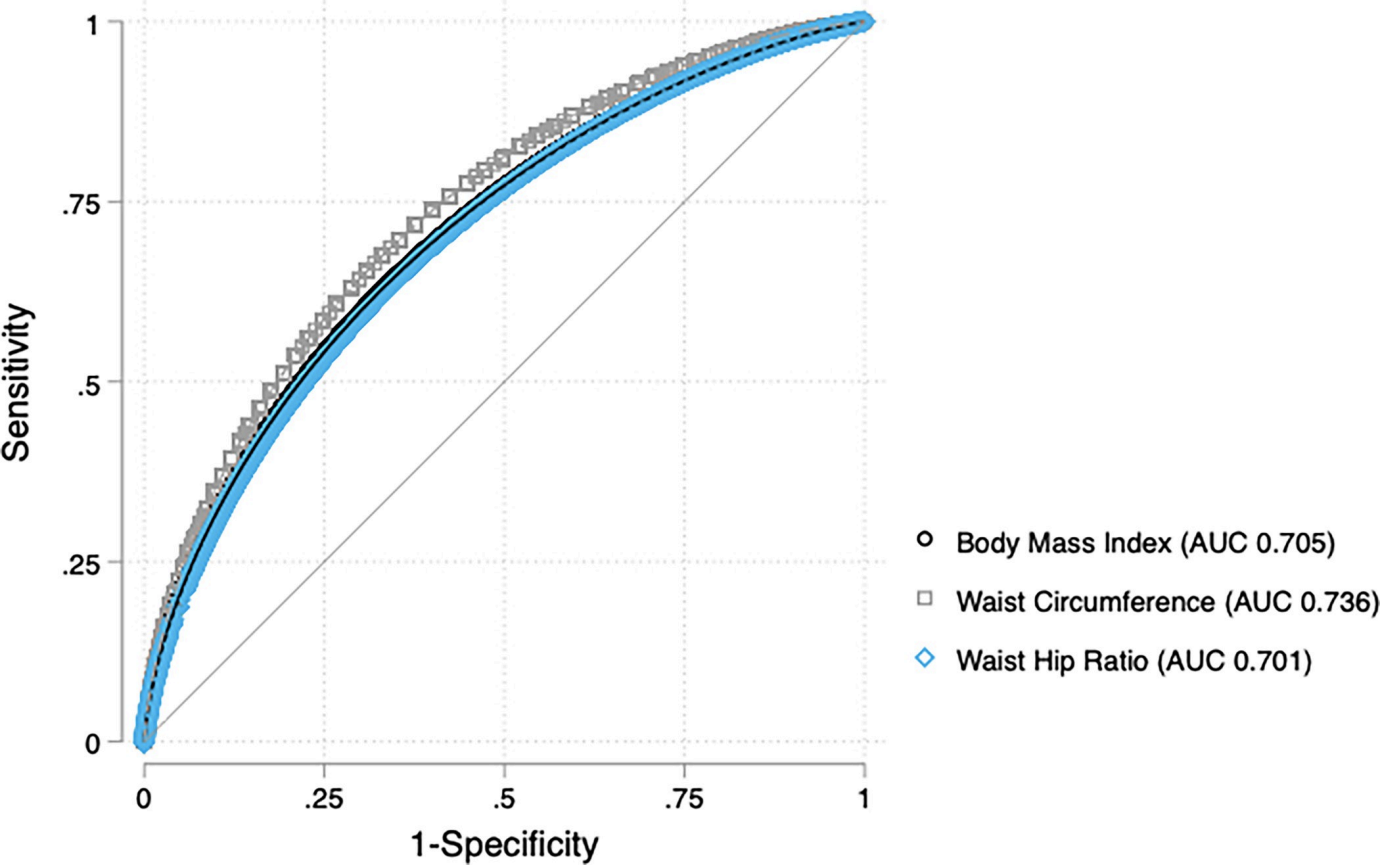
Anthropometric ROC curves for dysglycemia in women. Receiver Operating Characteristic (ROC) Curves for Body Mass Index, Waist Circumference, and Waist Hip Ratio to detect dysglycemia. For women, the AUC for each anthropometric measure was 0.705 for BMI (95% CI 0.692–0.719), 0.736 for Waist Circumference (95% CI 0.721–0.750), and 0.700 for Waist Hip Ratio (95% CI 0.686–0.715).

We compared the diagnostic validity of each anthropometric measure to detect dysglycemia using weighted parametric regression analyses of ROC curves. For men, WC had a greater area under the curve compared to WHR (WC area under the curve (AUC) 0.774 [95% CI 0.744–0.805] vs. WHR AUC 0.707 [95% CI 0.674–0.739], p-value <0.001). However, the discriminatory capacity was similar between WC and BMI among men (WC AUC 0.774 [95% CI 0.744–0.805] vs. BMI AUC 0.768 [95% CI 0.739–0.797], p-value = 0.589, Fig 1). Among women, WC had the highest discriminatory capacity compared to BMI (WC AUC 0.736 [95% CI 0.721–0.750] vs. BMI AUC 0.705 [95% CI 0.692–0.719], p-value<0.001) and WHR (WHR AUC 0.700 [95% CI 0.686–0.715], p-value<0.001, Fig 2). BMI had a greater AUC compared to WHR only for men (men p-value = 0.001, women p-value = 0.589).

When assessing the traditional anthropometric thresholds defined by the WHO, NHLBI, and IDF, we found reasonably high sensitivity and specificity for detecting dysglycemia among women (Table 2). For example, the sensitivity for WC >80 cm to detect dysglycemia was 91.1% (95% CI 89.5–92.5) with a corresponding specificity of 31.3% (95% CI 30.4–32.2; positive predictive value 14.9%). By contrast, traditional thresholds recommended for men resulted in much lower sensitivity such that a WC >94 cm to detect dysglycemia yielded a sensitivity of 53.5% (95% CI 47.8–59.1) and specificity of 88.5% (95% CI 87.6–89.3; positive predictive value 21.1%).

**Table 2:** Traditional anthropometric thresholds.

Traditional Thresholds Applied in our Study Population							
Men				Women			
Threshold Criterion	Sensitivity (%)	Specificity (%)	PPV^#^	Threshold Criterion	Sensitivity (%)	Specificity (%)	PPV^#^
**Body Mass Index (kg/m2)**							
WHO^†^ ≥25	74 (68.8–78.7)	72.8 (71.6–73.9)	13.6	WHO^†^ ≥25	91 (89.4–92.4)	34.4 (33.5–35.3)	15.5
WHO^†^ ≥30	41.3 (35.8–46.9)	90.7 (89.9–91.5)	20.4	WHO^†^ ≥30	72.5 (70.1–74.8)	59.4 (58.5–60.3)	19.1
**Waist Circumference (cm)**							
IDF^‡^ >94	53.5 (47.8–59.1)	88.5 (87.6–89.3)	21.1	IDF^‡^ >80	91.1 (89.5–92.5)	31.3 (30.4–32.2)	14.9
NHLBI^§^ >102	33.4 (28.2–39.0)	94.5 (93.8–95.1)	25.9	NHLBI^§^ >88	84.1 (82.1–85.9)	47.6 (46.7–48.6)	17.5
**Waist Hip Ratio**							
WHO^¶^ > 0.9	61.5 (55.9–67.0)	74.6 (73.4–75.8)	12.2	WHO^¶^ > 0.85	75.2 (72.8–77.4)	56.2 (55.3–57.2)	18.4

Sensitivity and Specificity with 95% Confidence Intervals.

^†^ WHO thresholds for overweight (≥25 kg/m^2^) and obese (≥30 kg/m^2^).

^‡^ International Diabetes Federation (IDF) waist circumference thresholds for cardiometabolic disease risk in women (>80 cm) and men (>94 cm).

^§^ NHLBI waist circumference thresholds for cardiometabolic disease risk in women (>88 cm) and men (>102 cm).

^¶^ WHO waist-hip ratio thresholds for cardiometabolic disease risk in women (>0.85) and men (>0.9).

^#^PPV (Positive Predictive Value) is the probability an individual has the disease following a positive test.

Given the poor performance of the international thresholds for WC in the men in this population, we derived population-specific thresholds to optimize sensitivity (>90%), specificity (>60%) and to balance both (Youden index) (Table 3) [31]. Applying the derived WC threshold with minimum sensitivity of 90%, we found that 73% of men in this population would screen positive and meet criteria for additional biochemical testing, however of those screened only 6.7% would have the disease (positive predictive value 6.7%). Alternatively, if we apply the WC threshold derived from a specificity >60% (i.e., WC >79.5cm), then fewer men in the population would require dysglycemia testing (39%) and the positive predictive value would increase (10.4%). The Youden Index WC threshold (>85.8cm) yielded a sensitivity of 69.4%, a specificity of 75.3%, and the highest positive predictive value of 13.9% (Table 3).

**Table 3:** Derived thresholds for each anthropometric measure based on sensitivity, specificity, and youden index.

**Derived Thresholds by Waist Circumference**							
**Males**				**Females**			
Threshold Criterion (Derived Cutoff)	Sensitivity (%)	Specificity (%)	PPV (%)	Threshold Criterion (Derived Cutoff)	Sensitivity (%)	Specificity (%)	PPV (%)
*Sensitivity 90%*	90.4 (86.6–93.5)	26.9 (25.7–28.1)	6.7	*Sensitivity 90%*	90.5 (88.8–92.0)	33.4 (32.5–34.3)	15.2
(> 72 cm)				(> 81 cm)			
*Specificity* *≥* *60*	78.0 (73.0–82.5)	61.1 (59.8–62.4)	10.4	*Specificity* *≥* *60*	75.1 (72.7–77.3)	60.0 (59.1–61.0)	19.9
(> 79.5 cm)				(> 93 cm)			
*Youden Index*	69.4 (64.0–74.5)	75.3 (74.1–76.4)	13.9	*Youden Index*	67.6 (65.1–70)	69.2 (68.4–70.1)	22.5
(> 85.8 cm)				(> 97.8 cm)			
**Derived Thresholds by BMI**							
**Males**				**Females**			
Threshold Criterion (Derived Cutoff)	Sensitivity (%)	Specificity (%)	PPV (%)	Threshold Criterion (Derived Cutoff)	Sensitivity (%)	Specificity (%)	PPV (%)
*Sensitivity 90%*	90.1 (86.2–93.1)	38.4 (37.1–39.7)	7.8	*Sensitivity 90%*	90.2 (88.6–91.7)	35.4 (34.5–36.3)	15.5
(> 21.2 kg/m2)				(> 25.2 kg/m2)			
*Specificity* *≥* *60*	84.6 (80.1–88.4)	59.2 (57.9–60.6)	10.7	*Specificity* *≥* *60*	72.2 (69.8–74.6)	59.5 (58.5–60.4)	18.9
(> 23.2 kg/m2)				(> 30.0 kg/m2)			
*Youden Index*	72.4 (67.1–77.3)	75.0 (73.8–76.2)	14.3	*Youden Index*	75.0 (72.6–77.2)	56.8 (55.8–57.7)	18.5
(> 25.3 kg/m2)				(> 29.4 kg/m2)			
**Derived Thresholds by Waist Hip Ratio**							
**Males**				**Females**			
Threshold Criterion (Derived Cutoff)	Sensitivity (%)	Specificity (%)	Positive Predictive Value (%)	Threshold Criterion (Derived Cutoff)	Sensitivity (%)	Specificity (%)	Positive Predictive Value (%)
*Sensitivity 90%*	90.1 (86.2–93.1)	26.0 (24.9–27.2)	6.6	*Sensitivity 90%*	90.5 (88.8–91.9)	28.5 (27.6–29.4)	14.2
(> 0.80)				(> 0.78)			
*Specificity* *≥* *60*	69.9 (64.4–74.9)	62.1 (60.8–63.4)	9.6	*Specificity* *≥* *60*	71.4 (69.0–73.8)	60.3 (59.4–61.3)	19.1
(> 0.87)				(> 0.86)			
*Youden Index*	55.1 (49.4–60.7)	80.7 (79.7–81.8)	14.1	*Youden Index*	67.9 (65.4–70.4)	64.2 (63.3–65.1)	19.9
(> 0.92)				(> 0.87)			

PPV (Positive Predictive Value) is the probability an individual has the disease following a positive test cm (centimeter); kg (kilogram).

**Fig 3:**
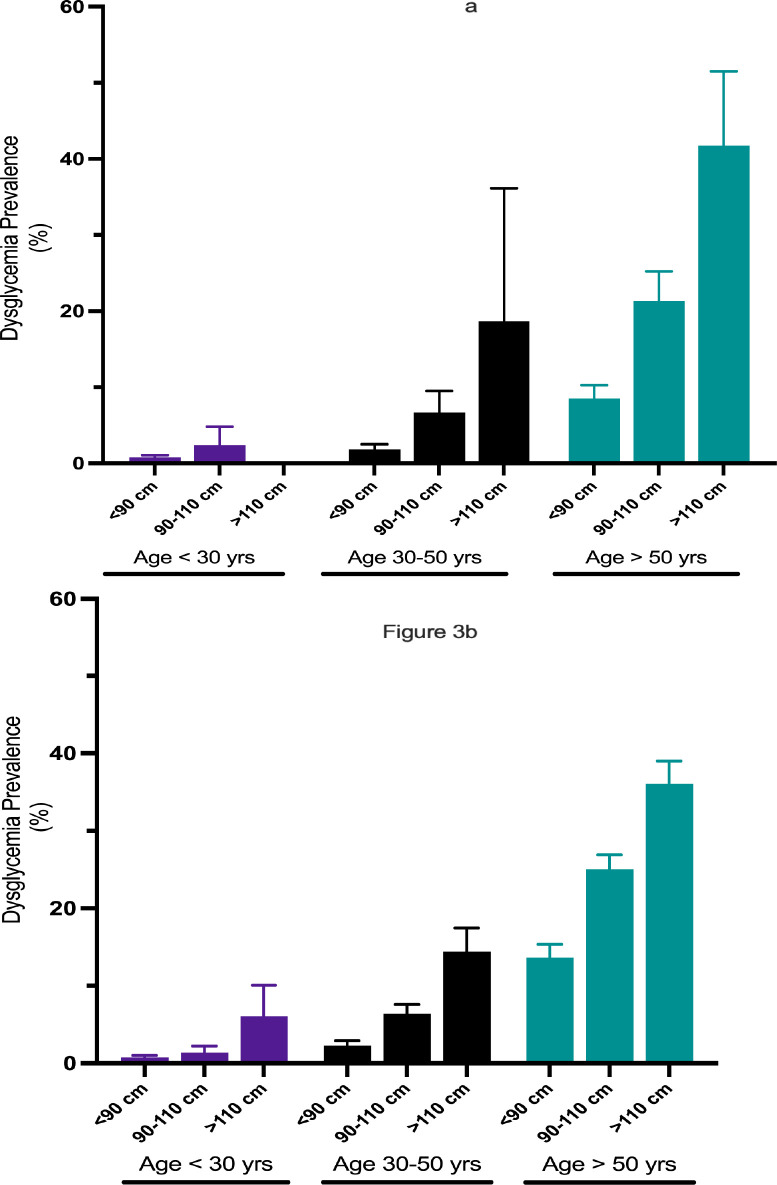
Prevalence of dysglycemia by waist circumference, stratified by sex. Fig 3A (men) and Fig 3B (women) demonstrate the prevalence of dysglycemia by age and waist circumference categories. This figure highlights the importance of age on dysglycemia for any given waist circumference category.

In both the univariate and multivariable logistic regression analyses, several variables were identified as significant predictors of dysglycemia (S1 Table). Specifically, being female, aged over 50, and having HIV-negative status were significantly associated with higher odds of dysglycemia in models that used WC and WHR as predictor variables. In contrast, in the BMI model, only age over 50 and HIV-negative status were significant predictors of increased odds of dysglycemia (S1 Table). We also observed significant two-way interactions between sex, age, and each of the anthropometric measures—WC, WHR, and BMI (S2 Table, S1 Fig). To illustrate, the predicted prevalence of dysglycemia among young women (under 30 years old) with a WC greater than 110 cm was 6.0% (95% CI: 2.0–10.1). This is markedly lower than the predicted prevalence of 36.0% (95% CI: 33.0–39.0, p-value <0.001) among women aged over 50 with the same WC (Fig 3). Additionally, our analyses indicated a sex-specific interaction in dysglycemia prevalence based on WC. Specifically, women had higher dysglycemia prevalence than men until the WC exceeded 130 cm. Beyond this point, men showed a higher predicted prevalence of dysglycemia (S2 Table and S1 Fig). Interestingly, when stratified by disease control, HIV status did not significantly interact with any of the anthropometric measures—BMI, WC, or WHR—in predicting dysglycemia (S2 Table).

## Discussion

Using a comprehensively characterized population-based sample of 17,846 individuals in sub-Saharan Africa, we identified that WC was the optimal anthropometric measure to screen for dysglycemia. This finding is consistent with prior data that have determined WC outperforms BMI and WHR in Asian and Western populations [34]. Furthermore, we found that IDF-recommended WC thresholds for women (>80cm) had high sensitivity and reasonable positive predictive values for the detection of disease, confirming this threshold would be an appropriate diabetes screening tool for this population. By contrast, IDF-recommended thresholds for men (>94 cm) had relatively low sensitivity (i.e., sensitivity 53%). We found all derived male WC thresholds were lower than the IDF threshold of 94 cm regardless of whether these were based on a sensitivity >90%, specificity >60%, or the Youden index.

Our results highlight important themes regarding the use of body anthropometrics as metabolic screening tools in this population. First, these data suggest it is time to move beyond BMI and start routinely measuring WC to assess for dysglycemia. Health professionals would need to be trained to properly perform this simple measurement, but in accordance with Ross *et al*., we recommend incorporating WC as a routine “vital sign” in clinical practice [35]. Second, we found that men have smaller WC thresholds to detect >90% of prevalent disease compared to women, which is an inverse pattern from that reported in European, US, and Asian populations [36]. Similar inverse relationships have been demonstrated in other anthropometric studies from Sub-Saharan Africa [13,16,17,37,38]. There are several potential factors that may explain these differences: regional variation of visceral adiposity [39], cultural dietary habits, and male-predominant occupations involving physical activity [40] that result in lower dysglycemia prevalence among men. We explored whether poorly controlled HIV may be contributing to the lower thresholds observed in men, but contrary to our hypothesis, we did not find significant differences in the anthropometric-metabolic disease relationship by HIV serostatus. Our results reinforce the need for further regional study of anthropometrics and dysglycemia to elucidate why South African men have comparatively lower anthropometric thresholds for dysglycemia than South African women and other ethnic groups.

Policy makers and guideline authors must weigh the advantages and disadvantages of selecting screening criteria to prioritize sensitivity or specificity. For example, if one selects the WC threshold with sensitivity >90%, approximately 73% of men would screen positive with a relatively low percentage having disease on confirmed serologic testing (PPV 6.7%). For a condition like diabetes, where the vast majority of those are undiagnosed in both KwaZulu-Natal and South Africa(24), adopting thresholds with high sensitivity (and thus lower specificity) would help promote enhanced detection of disease to better achieve programmatic goals set by the WHO’s *Global Diabetes Compact*. Notably, implementation would also require adequate resources to respond to the high detection rate (e.g., testing capacity and referral services). By contrast, in settings with severely restrained resources, it may be more feasible to select screening criteria with higher specificity (and lower sensitivity) to target resources to persons most likely to have disease. For example, if one prioritizes criteria with a specificity >60% (≥79.5 cm), then approximately 39% of men would screen positive, prompting confirmatory diabetes testing and yielding a positive predictive value of 10.4%. As such, threshold selection should be conducted alongside health systems’ preparedness and cost-effective assessments to help ensure applying thresholds translates into an optimal public health response. Importantly, positive predictive values for men should not be directly compared to the positive predictive values of women in our results, as both sample size and prevalence of diabetes differ between the two groups.

We suspect that some traditional anthropometric thresholds performed poorly in this rural African population because they were derived to detect obesity, not dysglycemia, and were generated from different populations. For example, the NHLBI data includes Caucasian adults, among whom a WC of 102 cm in men and 88 cm in women corresponded to the BMI threshold of obesity (30.0 kg/m^2^). Similarly, the IDF used cross-sectional European data to generate WC and WHR thresholds to correspond to BMI of ≥25.0 kg/m^2^ [41]. These thresholds were not generated based on associations with metabolic disease, but instead meant to be an alternative measure of obesity [29,35]. Our study used prevalence of dysglycemia as the outcome of interest and we prioritized high sensitivity thresholds with positive predictive value to optimize disease screening. Furthermore, in most populations, the prevalence of obesity is greater for women than for men. However, the magnitude of the difference between the sexes varies greatly by country such that in the U.S., female obesity prevalence exceeds that of men by 4%. By contrast, in South Africa, the female excess is 29% [42]. We found a similar prevalence of excess obesity in women in our cohort [43–45]. Lastly, the age distributions between our target population and those used to derive the traditional thresholds differ. For example, the Netherlands population used to validate the IDF thresholds for metabolic disease included 2,183 men with an average age of 42.7 years (SD 10.5) and 2,698 women aged 42.5 years (SD 10.7) [46]. By contrast, our study population had 5,735 men with an average age of 33.6 years (SD 15.1) and 12,111 women aged 40.2 years (SD 19.9). Although the ages were younger in our cohort, our study reflects the demographic and age structure of South Africa, where both life expectancy and mean age are approximately 15 years lower than in the Netherlands [20,47].

## Strengths and limitations

Our study was strengthened by a large study population size and inverse probability weights for non-response to better represent the base population characteristics. There were also important limitations. The cross-sectional design prevents the ability to make casual inferences between anthropometric measures and dysglycemia. Importantly, this also prevented us from using diabetes as a definitive outcome measure, which requires repeated testing over time [22]. Furthermore, there are conflicting data about the accuracy of hemoglobin A1c testing among certain subgroups that could affect the results of our study. Although not consistent across all African populations [48,49], hemoglobin A1c testing has been found to be falsely low in individuals living with HIV in certain studies [50,51]. Ideally, we would have used 2-hour oral glucose tolerance tests to characterize dysglycemia, but it is not feasible in large epidemiologic studies. Older women were more likely to participate in the health fair compared to men and younger individuals. Women in our study population have higher BMI, WC, and more than double the prevalence of dysglycemia, which could result in a confounding effect of age in our threshold analyses. We attempt to address the potential for selection bias by non-random sampling with the use of inverse probability sample weights.

## Conclusions

In conclusion, we found that WC outperforms BMI and WHR in detection of prevalent dysglycemia in this rural South African population. International Diabetes Federation WC thresholds for women (> 80 cm) may be used as a sensitive screening tool for dysglycemia, however optimal thresholds for screening men were lower compared to recommended guidelines. In low- and middle- income countries such as South Africa, it may be preferred to select screening criteria with higher specificity for a more cost-effective strategy. Finally, our results reinforce that locally derived data are needed to determine optimal screening and referral targets for dysglycemia.

## Supporting information

S1 Fig Predicted dysglycemia prevalence based on waist circumference interactions with sex and age. (DOCX)

S1 Table Univariable and multivariable logistic regression models for dysglycemia. (DOCX)

S2 Table Adjusted logistic regression models with interaction terms for dysglycemia. (DOCX)
