# Supplementary material for: Identifying sex-specific anthropometric measures and thresholds for dysglycemia screening in an HIV-endemic rural South African population
Source: PLOS Glob Public Health. 2023 Oct 27;3(10):e0001698. doi: 10.1371/journal.pgph.0001698 (PMC10610455; doi:10.1371/journal.pgph.0001698)

**S1 Fig: Predicted Dysglycemia Prevalence based on Waist Circumference Interactions with Sex and Age**


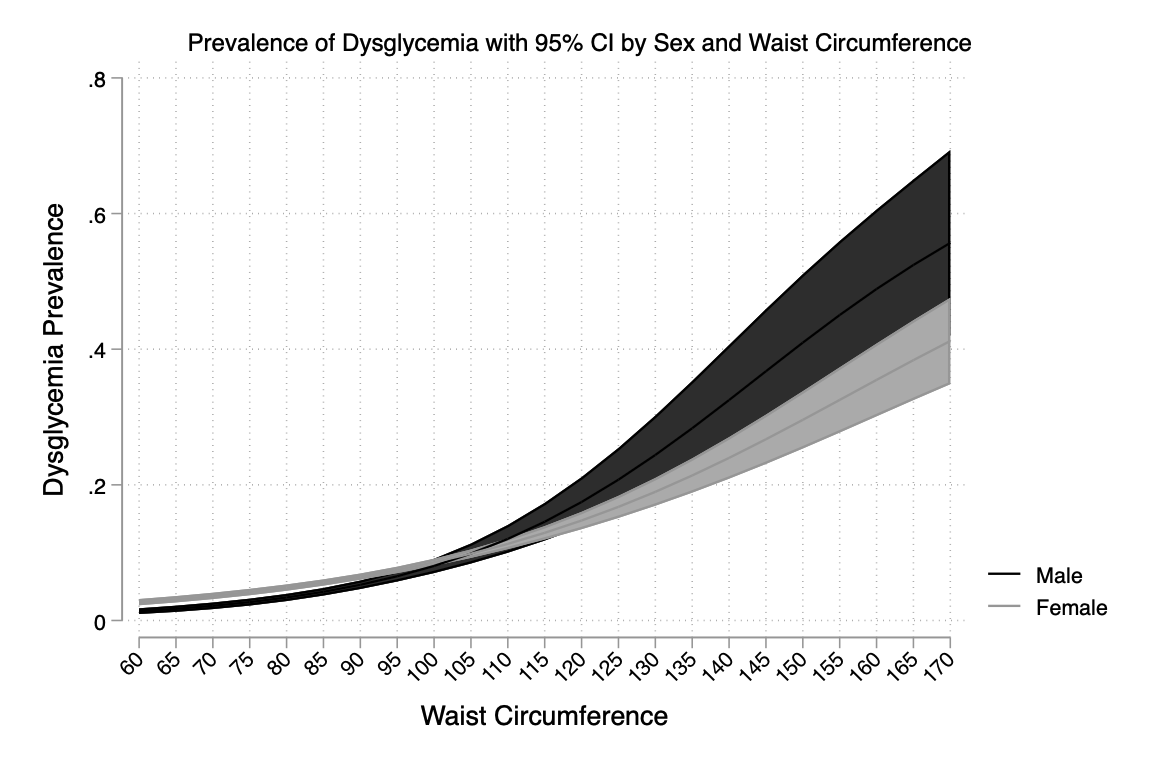


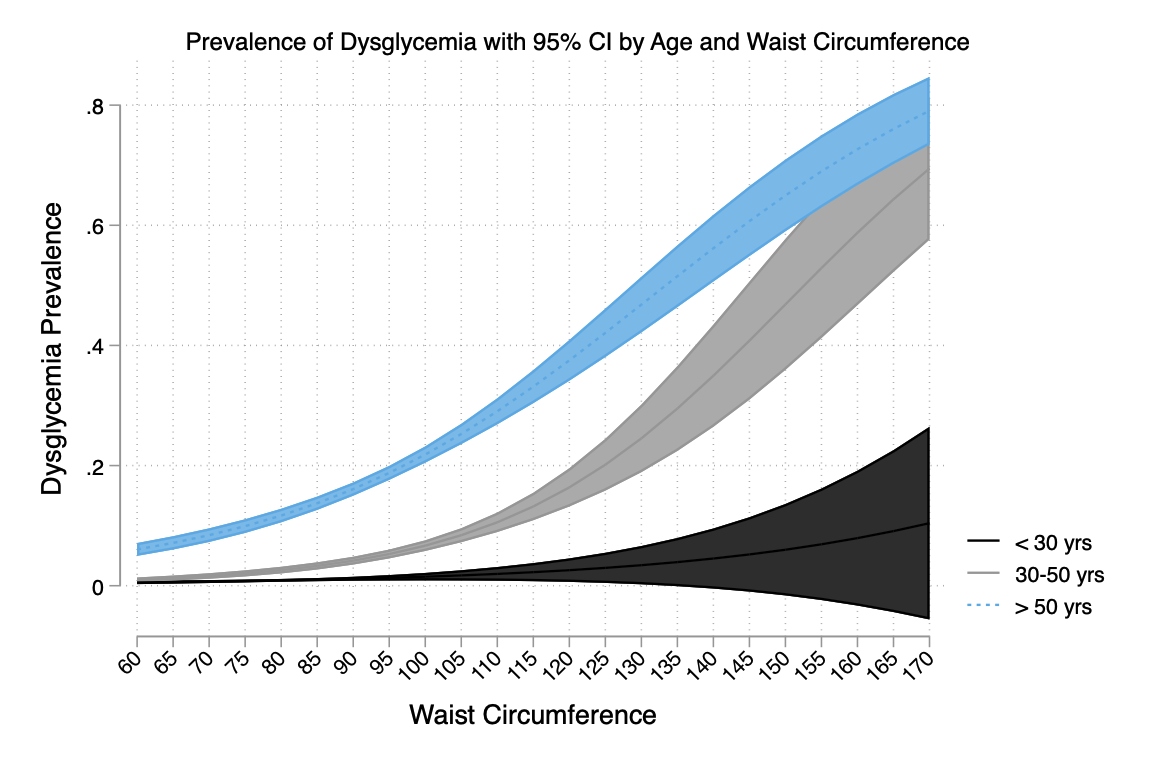

Supplement: S1 Fig — (DOCX) [file pgph.0001698.s001.docx]
