# Supplementary material for: Identifying sex-specific anthropometric measures and thresholds for dysglycemia screening in an HIV-endemic rural South African population
Source: PLOS Glob Public Health. 2023 Oct 27;3(10):e0001698. doi: 10.1371/journal.pgph.0001698 (PMC10610455; doi:10.1371/journal.pgph.0001698)
Supplement: S1 Table — (DOCX) [file pgph.0001698.s002.docx]

**S1 Table: Univariable and Multivariable Logistic Regression Models for Dysglycemia**

| **Characteristic** | **Univariable Odds Ratio (95%CI)** | **p-value** | **Adjusted Odds Ratio (95% CI)** | **p-value** |
| --- | --- | --- | --- | --- |
| BMI |  |  |  |  |
| Female | 2.5 (2.2-2.9) | <0.001* | 1.1 (1.0-1.3) | 0.161 |
| HIV-controlled | 0.6 (0.6-0.7) | <0.001* | 0.6 (0.5-0.7) | <0.001* |
| HIV- uncontrolled | 0.3 (0.2-0.5) | <0.001* | 0.6 (0.4-0.9) | 0.008* |
| Age <30 | 0.2 (0.1-0.3) | <0.001* | 0.2 (0.2-0.3) | <0.001* |
| Age >50 | 5.6 (4.9-6.5) | <0.001* | 4.4 (3.8-5.1) | <0.001* |
| BMI | 1.1 (1.1-1.1) | <0.001* | 1.1 (1.1-1.1) | <0.001* |
| Waist circumference | |  |  |  |
| Female | 2.5 (2.2-2.9) | <0.001* | 1.2 (1.0-1.4) | 0.018* |
| HIV-controlled | 0.6 (0.6-0.7) | <0.001* | 0.6 (0.5-0.7) | <0.001* |
| HIV- uncontrolled | 0.3 (0.2-0.5) | <0.001* | 0.6 (0.4-0.9) | 0.010* |
| Age <30 | 0.2 (0.1-0.3) | <0.001* | 0.3 (0.2-0.4) | <0.001* |
| Age >50 | 5.6 (4.9-6.5) | <0.001* | 4.0 (3.4-4.6) | <0.001* |
| WC | 1.1 (1.1-1.1) | <0.001* | 1.0 (1.0-1.1) | <0.001* |
| Waist Hip Ratio | |  |  |  |
| Female | 2.5 (2.2-2.9) | <0.001* | 2.0 (1.8-2.3) | <0.001* |
| HIV-controlled | 0.6 (0.6-0.7) | <0.001* | 0.5 (0.5-0.6) | <0.001* |
| HIV- uncontrolled | 0.3 (0.2-0.5) | <0.001* | 0.6 (0.4-0.8) | 0.002* |
| Age <30 | 0.2 (0.1-0.3) | <0.001* | 0.2 (0.2-0.3) | <0.001* |
| Age >50 | 5.6 (4.9-6.5) | <0.001* | 3.9 (3.4-4.5) | <0.001* |
| WHR | 483.1 (283-823) | <0.001* | 59.2 (34.0-103) | <0.001* |
| *p-value <0.05 |  |  |  |  |
