# Supplementary material for: Identifying sex-specific anthropometric measures and thresholds for dysglycemia screening in an HIV-endemic rural South African population
Source: PLOS Glob Public Health. 2023 Oct 27;3(10):e0001698. doi: 10.1371/journal.pgph.0001698 (PMC10610455; doi:10.1371/journal.pgph.0001698)
Supplement: S2 Table — (DOCX) [file pgph.0001698.s003.docx]

**S2 Table: Adjusted Logistic Regression Models with Interaction Terms for Dysglycemia**

| **Characteristic** | **Adjusted Odds Ratio (95% CI)** | **p-value** |
| --- | --- | --- |
| BMI |  |  |
| Female | 6.42 (3.27-12.61) | <0.001* |
| HIV-controlled | 0.49 (0.27-0.85) | 0.012* |
| HIV- uncontrolled | 0.36 (0.09-1.51) | 0.163 |
| Age <30 | 0.84 (0.27-2.59) | 0.03* |
| Age >50 | 10.01 (5.41-18.54) | <0.001* |
| BMI | 1.15 (1.12-1.19) | <0.001* |
| BMI*Sex | 0.94 (0.91-0.96) | <0.001* |
| BMI*HIV-controlled | 1.01 (0.99-1.03) | 0.355 |
| BMI*HIV-uncontrolled | 1.02 (0.98-1.06) | 0.387 |
| BMI*Age<30 | 0.96 (0.92-1.00) | 0.035* |
| BMI*Age >50 | 0.98 (0.96-0.99) | 0.006* |
| Waist Circumference |  |  |
| Female | 5.29 (2.06-13.55) | <0.001* |
| HIV-controlled | 0.25 (0.10-0.59) | 0.002* |
| HIV- uncontrolled | 1.15 (0.10-13.06) | 0.912 |
| Age <30 | 2.11 (0.33-13.5) | 0.431 |
| Age >50 | 13 (5.03-33.61) | <0.001* |
| WC | 1.06 (1.05-1.07) | 0.001* |
| WC*Sex | 0.98 (0.97-0.99) | 0.002* |
| WC*HIV-controlled | 1.01 (0.99-1.02) | 0.058 |
| WC*HIV-uncontrolled | 0.92 (0.97-1.02) | 0.555 |
| WC*Age<30 | 0.98 (0.96-0.99) | 0.035* |
| WC*Age >50 | 0.99 (0.98-0.99) | 0.011* |
| Waist Hip Ratio |  |  |
| Female | 2.51 (0.66-9.50) | 0.174 |
| HIV-controlled | 0.28 (0.8-0.97) | 0.045* |
| HIV- uncontrolled | 0.59 (0.02-17.2) | 0.758 |
| Age <30 | 5.43 (0.60-49.17) | 0.132 |
| Age >50 | 17.63 (4.81-64.63) | <0.001* |
| WHR | 245.53 (39.48-1526.9) | <0.001* |
| WHR*Sex | 0.78 (0.18-3.31) | 0.738 |
| WHR*HIV-controlled | 2.01 (0.52-7.86) | 0.314 |
| WHR*HIV-uncontrolled | 0.98 (0.02-42.95) | 0.990 |
| WHR*Age<30 | 0.02 (0.002-0.29) | 0.004* |
| WHR*Age >50 | 0.19 (0.04-0.79) | 0.022* |
| *p-value <0.05 | | |
| Reference: Male, HIV negative, Age 30-50 years | | |
